# Effect of short-term ambient temperature exposure on influenza A and B incidence: a time-series analysis of daily surveillance data in Kawasaki City, Japan

**DOI:** 10.1016/j.ijregi.2024.100479

**Published:** 2024-10-24

**Authors:** Keita Wagatsuma

**Affiliations:** 1 Division of International Health (Public Health), Graduate School of Medical and Dental Sciences, Niigata University, Niigata, Japan; 2 Institute for Research Administration, Niigata University, Niigata, Japan

**Keywords:** Influenza, Temperature, Epidemics, Climate change, Transmission

## Abstract

**Objectives:**

Although there is epidemiological evidence that ambient temperature affects influenza transmission, the relationship between temperature and specific influenza virus types remains inadequately studied. This study aimed to investigate the association between ambient temperature exposure and the incidence counts of influenza A and B viruses in Kawasaki City, Japan.

**Methods:**

Daily data on influenza A and B cases and meteorological variables were collected from Kawasaki City, Japan, spanning from March 2014 to December 2019. A time-series quasi-Poisson regression model, in conjunction with a distributed lag non-linear model, was utilized to explore the exposure–response relationship between short-term influenza risk and daily mean temperature.

**Results:**

The study encompassed 181,895 patients with influenza. Our findings indicated that cold temperatures significantly heightened the risk of influenza A and B virus infections. The overall cumulative relative risk of influenza A was 5.08 (95% confidence interval 3.64–7.08) at cold temperatures (5^th^ percentile, 5.6°C) compared with the median temperature (17.7°C) and 2.50 (1.82–3.44) for influenza B.

**Conclusions:**

This study suggests that the short-term effects of temperature on influenza transmission are type-specific. It offers crucial insights for future research on the complex associations between ambient temperature and respiratory infectious diseases.

## Highlights

• Ambient temperature and influenza A and B incidences in Japan were studied.
• The association between influenza incidence and temperature was non-linear.
• Lower temperature is associated with greater influenza risk for both virus types.
• Effects of temperature on influenza transmission are type-specific.

## Introduction

Influenza, an acute respiratory infection caused by the influenza virus, imposes a significant burden on global economies and societies [1]. Two primary types of influenza viruses, influenza A and influenza B, circulate widely and are responsible for seasonal epidemics. During the past few decades, studies have examined associations of influenza epidemics with meteorological factors, particularly ambient temperature, but the differential impact of influenza types has not been comprehensively evaluated [2]. Given the differing clinical severity and outcomes of influenza A and B viruses, as well as the fact that currently available influenza vaccines are tailored to specific types, it is essential to investigate the differences in the relationship between meteorological factors and influenza [1]. Herein, we conducted a time-series analysis of daily influenza surveillance data to explore the association between ambient temperature and the incidence counts of influenza types in Kawasaki City, Japan.

## Methods

Kawasaki City, located in Northeastern Kanagawa Prefecture adjacent to Tokyo, Japan, was selected as the study [3]. The city experiences a temperate climate with distinct seasons characterized by hot, humid summers and cold, dry winters. The Kawasaki City Infectious Disease Surveillance System collects real-time data on influenza infections. Since March 2014, all city medical facilities have been required to report daily influenza diagnoses. Rapid diagnostic test kits are employed to differentiate between influenza types A and B, with the daily surveillance data made available through a city-operated website [4]. For this study, daily total numbers of influenza A and B cases from March 2014 to December 2019 were obtained. We also obtained daily mean temperature (°C), relative humidity (%), wind speed (m/s), total rainfall (mm), and sunshine duration (hours) data for the same study period from the Japan Meteorological Agency, measured at a single weather station located in central Kawasaki City [5].

A time-series regression analysis was performed to investigate the relationship between daily ambient temperature and the incidence counts of influenza A and B viruses. The analysis employed a generalized linear model with a quasi-Poisson distribution and a logarithmic link function in conjunction with a distributed lag non-linear model. Non-linear and delayed effects were captured using a cross-basis function, modeled with a natural cubic B-spline for mean temperature, incorporating three internal knots at the 10^th^, 75^th^, and 90^th^ percentiles. Based on prior literature, lag periods extending up to 21 days were examined, utilizing a natural cubic spline with three internal knots distributed on a log scale [6]. We included a time period of 7 degrees of freedom (df) per year in the main model to control for seasonality and long-term trends. In addition, the main model controlled for a 21-day moving average of relative humidity (a natural cubic B-spline with 3 df), day of the week, and public holidays. We added an offset term for the number of medical facilities reporting each day to account for daily variations. Lastly, we included an autoregressive term for daily counts at lags of 1 and 2 days to align with the transmission mechanism. For sensitivity analysis, we tested different model selections by adjusting the df for time trends (5 and 9 df per year), using different lag periods (14 and 28 days), excluding relative humidity, and incorporating control for a 21-day moving average of wind speed, total rainfall, and sunshine duration (a natural cubic spline with 3 df). We then estimated the lag-cumulative relative risks (RRs) and their 95% confidence intervals for cold (5^th^ percentile) and heat (95^th^ percentile) compared with the 50^th^ percentile of mean temperature as a reference value. All statistical analyses were conducted using R 3.5.2 (R Core Team, R Foundation for Statistical Computing, Vienna, Austria), specifically using the *dlnm* package.

## Results

**Figure 1:**
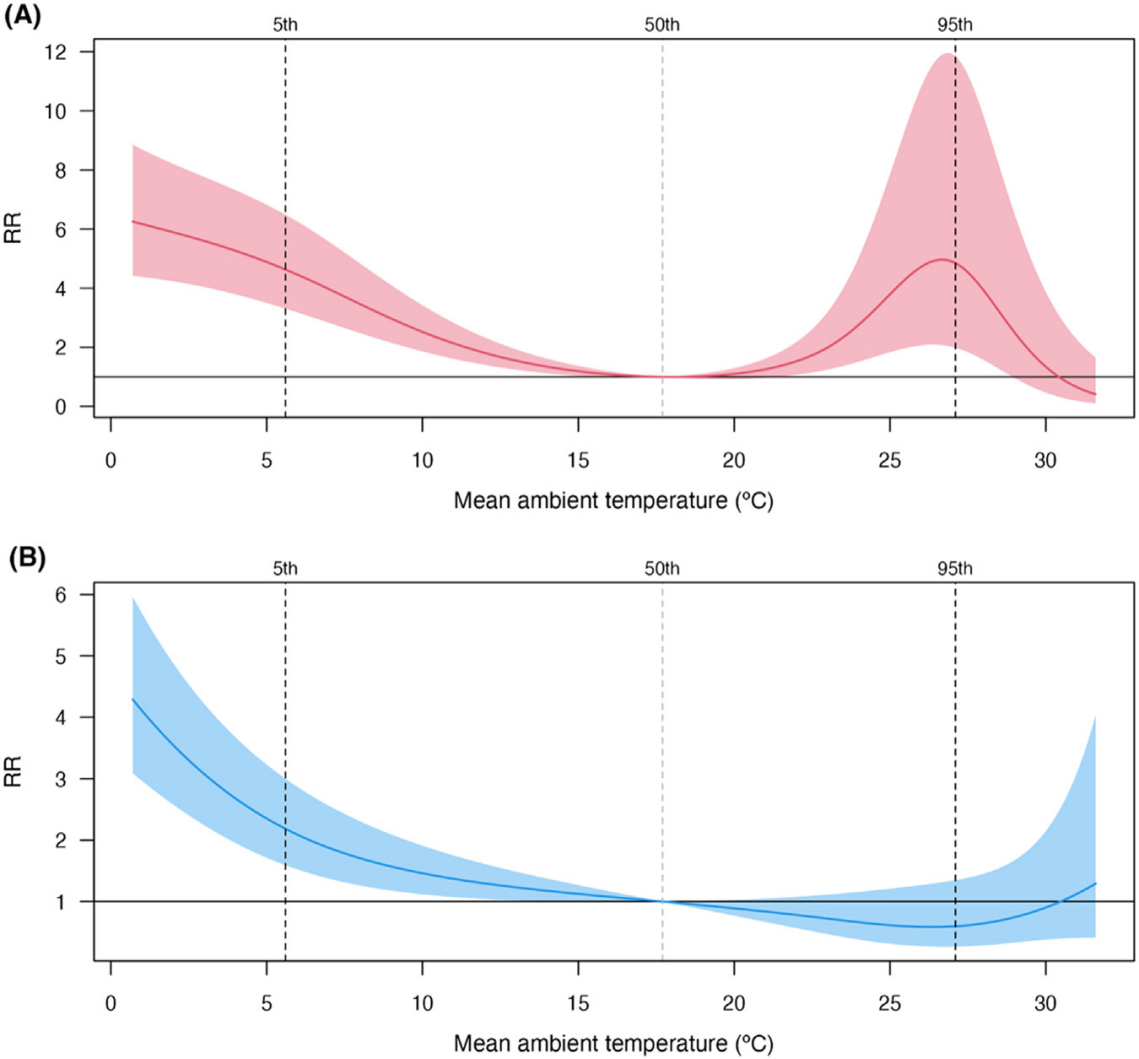
Associations between daily mean ambient temperature and incidence counts of influenza A and B viruses. The figure depicts the estimated curves showing the associations between daily mean temperature (with 95% confidence intervals, shaded region) and the incidence counts of influenza A (A) and influenza B (B) viruses. The dotted vertical line indicates the daily mean ambient temperature of 17.7 °C, corresponding to the 50^th^ percentile of temperature, whereas the dashed vertical lines represent the 5^th^ and 95^th^ percentiles of temperature (5.6 °C and 27.1 °C). RR, relative risk.

Between March 2014 and December 2019, a total of 181,895 influenza cases were observed in Kawasaki City. Of these, 131,726 cases (72.4%) were attributed to influenza A, whereas 50,169 cases (27.6%) were attributed to influenza B. Summary statistics are provided in Table S1. A time-series plot of daily reported influenza A and B cases indicated that seasonal peaks varied annually but generally occurred during the winter months (Figure S1). The number of both influenza A and B cases was negatively correlated with mean temperature (*ρ* = –0.80 and –0.63, respectively) (Table S2). Figure 1 presents an exposure–response curve illustrating the overall cumulative association between daily mean ambient temperature and the incidence counts of influenza A and B viruses over a 0-21 day lag period. The curve shapes differed depending on the influenza subtype: influenza A exhibited a bimodal M-shaped curve, whereas the risk of influenza B decreased monotonically with increasing temperature. For influenza A, the overall cumulative RRs for cold (5^th^ percentile 5.6 °C) and heat (95^th^ percentile 27.1 °C) were 5.08 (95% confidence interval 3.64-7.08) and 4.85 (1.98–11.85), respectively, compared with the 50^th^ percentile (17.7 °C). In contrast, the RRs for influenza B were lower, with estimates of 2.50 (1.82–3.44) for cold and 0.59 (0.26-1.34) for heat relative to the 50^th^ percentile (17.7 °C). The sensitivity analysis revealed that varying the choice of model had little effect on the estimates (Table S3). Nonetheless, the observed increased risk of influenza A at high temperatures was not consistently present across all models, indicating a lack of stability.

## Discussion

In recent years, we have seen extensive research on the effect of ambient temperature on respiratory infections globally. However, the exposure–response relationship between ambient temperature and influenza incidence counts, particularly at the virus subtype level, remains underexplored. This study is the first in Japan to assess the association between ambient temperature and influenza incidence by virus subtype. Our analysis indicated that cold weather is associated with increased cumulative risks of influenza A and B virus activity, consistent with previous studies in temperate and subtropical regions [7]. Low temperatures (4 °C) enhance the stability of the influenza virus by promoting lipid ordering on the viral membrane, a process critical for airborne transmission [8]. The increased tendency for indoor activity during cold weather further elevates the contact rate [9]. Certain modeling choices also revealed another peak in influenza A incidence counts at high temperatures, consistent with reports of semiannual epidemics in subtropical Chinese cities during summer, though further research is required to elucidate the underlying mechanisms [10]. A plausible explanation for this mechanism is that in times experiencing relatively high outdoor temperatures, individuals are inclined to remain indoors with increased use of air conditioning, which may inadvertently enhance interpersonal contact and thus promote the transmission of influenza [11].

There are several limitations to this study. First, we used the prefecture level as a surrogate of personal exposure, which could induce measurement error. This exposure measurement error, known as Berkson error, may reduce the precision or power of effects. Second, the analysis is confined to data from Kawasaki City, necessitating the validation of the models with data from other regions. Finally, the findings may be prone to ecological fallacies; therefore, future research should employ more precise data or experimental designs.

In conclusion, our study determined that lower ambient temperatures are associated with an increased risk of influenza A and B virus infections in Kawasaki City, Japan. Notably, this study also suggests that the short-term effects of temperature on influenza transmission are type-specific. We recommend that ambient temperature be carefully considered in influenza prediction models in Japan, given the complex non-linear associations across different virus types. The findings highlight the critical role of monitoring and managing ambient temperature as a preventive measure against influenza. Enhanced comprehension of the environmental determinants influencing influenza transmission, segmented by type, would have critical implications for advancing the design of targeted and efficacious interventions against influenza.

## Declarations of competing interest

The authors have no competing interests to declare.

## Appendix Supplementary materials

Image, application 1
