# Supplementary material for: Effect of short-term ambient temperature exposure on influenza A and B incidence: a time-series analysis of daily surveillance data in Kawasaki City, Japan
Source: IJID Reg. 2024 Oct 24;13:100479. doi: 10.1016/j.ijregi.2024.100479 (PMC11609664; doi:10.1016/j.ijregi.2024.100479)
Supplement: Supplementary file 1 [file mmc1.docx]

**Short Communication**

**Title: Effect of short-term ambient temperature exposure on influenza A and B incidence: a time-series analysis of daily surveillance data in Kawasaki City, Japan**

**Authors:**

Keita Wagatsuma^a,b^

**Affliations:**

^a^ Division of International Health (Public Health), Graduate School of Medical and Dental Sciences, Niigata University, Niigata, Japan

^b^ Institute for Research Administration, Niigata University, Niigata,Japan

**Corresponding author:**

Keita Wagatsuma

Division of International Health (Public Health)

Graduate School of Medical and Dental Sciences, Niigata University

1-757 Asahimachi dori, Chuo-ku, Niigata City, Niigata 951-8510, Japan

Tel: +81-25-227-2129; Fax +81-25-227-0765

E-mail address: [waga@med.niigata-u.ac.jp](mailto:waga@med.niigata-u.ac.jp)

**
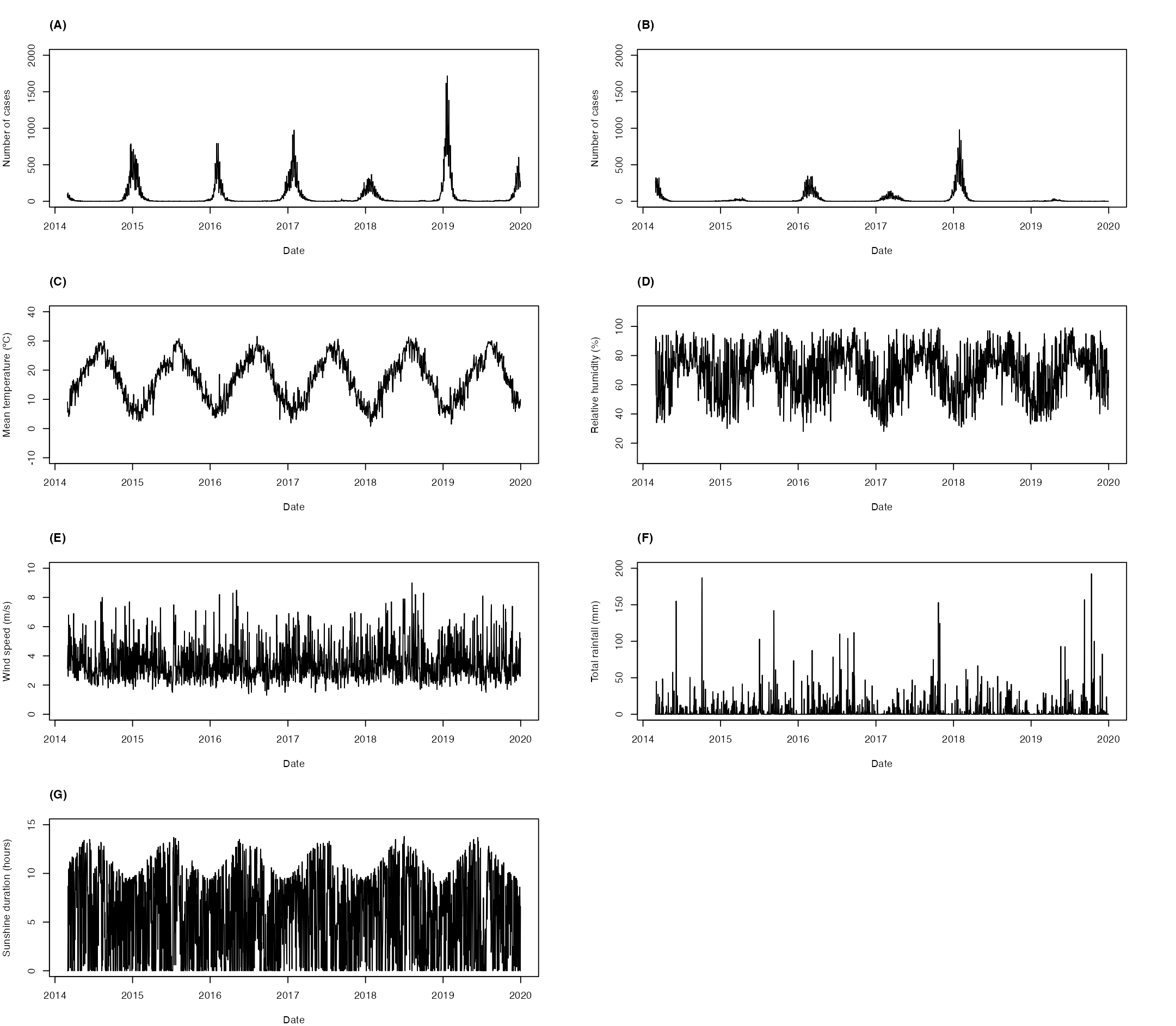
**

**Figure S1. Daily time-series of number of influenza A and B cases and meteorological variables.** The daily seasonal trends are illustrated as follows: (A) number of influenza A cases, (B) number of influenza B cases, (C) mean temperature (℃), (D) relative humidity (%), (E) wind speed (m/s), (F) total rainfall (mm), and (G) sunshine duration (hours) spanning from March 2014 to December 2019.

**Table S1. Summary statistics of daily number of influenza A and B cases and meteorological variables.**

| **Variables** | **Number of cases, n (%)** | **Minimum** | **Maximum** | **Mean** | **SD** |
| --- | --- | --- | --- | --- | --- |
| Influenza A | 131,726 (72.4) | 0 | 1,717 | 61.7 | 153.6 |
| Influenza B | 50,169 (27.6) | 0 | 981 | 23.5 | 74.7 |
| Mean temperature (℃) | – | 0.7 | 31.6 | 17.0 | 7.4 |
| Relative humidity (%) | – | 28.0 | 99.0 | 68.5 | 15.7 |
| Wind speed (m/s) | – | 1.3 | 9.0 | 3.4 | 1.9 |
| Total rainfall (mm) | – | 0.0 | 192.5 | 4.9 | 15.1 |
| Sunshine duration (hours) | – | 0.0 | 13.8 | 5.7 | 4.2 |

Abbreviations: SD, standard deviation.

**Table S2. Spearman’s rank-order cross-correlation coefficients between number of influenza A and B cases and meteorological variables.**

| **Variables** | **Influenza A** | **Influenza B** | **Mean temperature** | **Relative humidity** | **Wind speed** | **Total rainfall** | **Sunshine duration** |
| --- | --- | --- | --- | --- | --- | --- | --- |
| Influenza A | 1.00 |  |  |  |  |  |  |
| Influenza B | 0.58^***^ | 1.00 |  |  |  |  |  |
| Mean temperature | –0.80^***^ | –0.63^***^ | 1.00 |  |  |  |  |
| Relative humidity | –0.47^***^ | –0.39^***^ | 0.49^***^ | 1.00 |  |  |  |
| Wind speed | 0.09 | 0.14 | –0.09 | –0.14 | 1.00 |  |  |
| Total rainfall | –0.11 | 0.10 | 0.05 | 0.64 | 0.08 | 1.00 |  |
| Sunshine duration | –0.01 | 0.07 | 0.10 | –0.56 | 0.05 | 0.60 | 1.00 |

^***^P<0.05; ^***^P<0.01; ^***^P<0.001.

**Table S3. Sensitivity analysis.**

|  | **Pooled cumulative relative risks (with 95 % confidence intervals)** | | | | |
| --- | --- | --- | --- | --- | --- |
|  | **Influenza A** | | **Influenza B** | | |
|  | **Cold risk^a^** | **Heat risk^a^** | | **Cold risk^a^** | **Heat risk^a^** |
| Modelling choices |  |  | |  |  |
| Main model | 5.08 (3.64, 7.08) | 4.85 (1.98, 11.85) | | 2.50 (1.82, 3.44) | 0.59 (0.26, 1.34) |
| Df/year for seasonal control: 5 | 3.55 (2.61, 4.82) | 1.40 (0.77, 2.56) | | 2.12 (1.62, 2.78) | 0.41 (0.21, 0.80) |
| Df/year for seasonal control: 9 | 4.69 (3.04, 7.24) | 0.66 (0.25, 1.75) | | 2.35 (1.67, 3.32) | 0.38 (0.16, 0.92) |
| Lag choices |  |  | |  |  |
| Main model | 5.08 (3.64, 7.08) | 4.85 (1.98, 11.85) | | 2.50 (1.82, 3.44) | 0.59 (0.26, 1.34) |
| 14 days | 1.71 (1.31, 2.23) | 1.80 (0.85, 3.81) | | 2.33 (1.85, 2.94) | 0.70 (0.37, 1.33) |
| 28 days | 8.90 (5.82, 13.60) | 3.02 (1.06, 8.54) | | 1.59 (1.08, 2.35) | 0.57 (0.21, 1.56) |
| Control for relative humidity |  |  | |  |  |
| Main model | 5.08 (3.64, 7.08) | 4.85 (1.98, 11.85) | | 2.50 (1.82, 3.44) | 0.59 (0.26, 1.34) |
| Without relative humidity | 4.71 (3.38, 6.57) | 6.48 (2.79, 15.00) | | 2.06 (1.52, 2.79) | 0.51 (0.23, 1.13) |
| Control for wind speed, total rainfall, and sunshine duration |  |  | |  |  |
| Main model | 5.08 (3.64, 7.08) | 4.85 (1.98, 11.85) | | 2.50 (1.82, 3.44) | 0.59 (0.26, 1.34) |
| With wind speed, total rainfall, and sunshine duration | 4.73 (3.32, 6.73) | 5.34 (2.12, 13.46) | | 2.30 (1.60, 3.31) | 0.72 (0.28, 1.80) |

Abbreviations: Df, degrees of freedom.

^a^ The cold and heat risks are the relative risks at the 5^th^ (cold) and 95^th^ (heat) percentiles of daily mean ambinet temperature.
